# Supplementary material for: Subpopulations in Strains of Staphylococcus aureus Provide Antibiotic Tolerance
Source: Antibiotics (Basel). 2023 Feb 17;12(2):406. doi: 10.3390/antibiotics12020406 (PMC9952555; doi:10.3390/antibiotics12020406)
Supplement: Supplementary file 1 [file antibiotics-12-00406-s001.zip › antibiotics-2217150-supplementary.pdf]

Supplementary Information

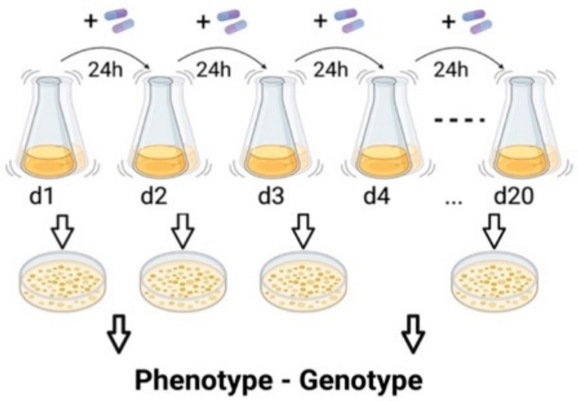

Figure S1. A schematic for Adaptive Laboratory Experiment (ALE) procedures. Antibiotic is added to fresh culture each day, and the bacteria (*S. aureus* UA-DI-55) is re-cultured each day, and samples taken (plates to observe colony types, SCV development, and other phenotypic characteristics and to assess genotypic changes).

File S1: Materials and Methods

Strains and culture media. The *S. aureus* clicnial isolate described in this study was sourced from the Royal Adelaide Hospital. The human ethics for this study was approved by the Human Research Ethics Committee at the Royal Adelaide Hospital (Approval number: HREC/17/RAH/242). Patients with an infected foot ulcer were classified as DFI and these were further divided into those with only a foot ulcer/wound infection (DFI-W) and those with osteomyelitis (DFI-OM). Isolate *S. aureus* UA-DI-55 was from a DFI-OM patient. Chemically defined media (CDM) composition was adapted from media used for coagulase-negative staphylococci slime production [70].

Adaptive laboratory evolution (ALE). ALE protocols were adapted from methods previously described [71]. UA-DI-55 was cultured in 5mL CDM to log phase (OD_{600} ~0.2). 500µL of culture was inoculated into a 250mL glass conical flask of 50mL CDM with a sub-MIC concentration of ciprofloxacin (250µg/mL) and incubated at 37°C. Every 24 hours, 100µL of culture was serially diluted and plated to TSA to determine CFU/mL and colony phenotypes. 500µL of culture was aliquoted to a fresh flask of 50mL CDM with sub-MIC ciprofloxacin. The remaining culture was centrifuged at 4000rpm at 4°C and resuspended in 40% glycerol for storage at -80°C.

Growth kinetics. Growth kinetics assays were performed in 96-well microtitre plates. Cells were incubated in TSB at 37°C to log phase (OD_{600} ~ 0.2). 20µL of log phase culture was added to a well containing 180µL of media. Culture was incubated at 37°C for 18 hours and OD_{600} readings taken every 30 minutes with a Sunrise Absorbance Microplate Reader (Tecan).

Minimum bacteriostatic and bactericidal concentrations of antibiotics. Antibiotics were serially diluted into TSB by a factor of 1:2. Cells were incubated in TSB at 37°C to log phase (OD_{600} ~ 0.2). 20µL of log phase culture was added to wells containing 180µL of TSB with antibiotic. Cultures were incubated at 37°C for 18 hours and OD_{600} readings were taken. Cultures were serially diluted to determine CFU/mL. Minimum inhibitory concentration (MIC) was determined to be the lowest concentration of antibiotic which resulted in no visual bacterial growth and change in OD_{600}. Minimum bactericidal

concentration (MBC) was determined to be the lowest concentration of antibiotic which resulted in a decrease in CFU/mL.

Whole genome sequencing. Whole genomic DNA was extracted and purified using QIAGEN Genomic-tip 500/G columns (QIAGEN, Australia) according to manufacturer protocols. Quality and quantity of genomic DNA was determined using FEMTO Pulse (SA Pathology). Genomes were sequenced using an Oxford Nanopore MinION device. The MinION library construction and sequencing were performed by FISABIO University of Valencia's sequencing service, using the Oxford Nanopore PCR barcoding kit following the manufacturer's instructions. We combined NextSeq (Illumina) with Minion (Oxford Nanopore) technologies to obtain complete, fully-closed genomes. Briefly, an average of 3.7 million reads of 150 bp of length sequenced by NextSeq were combined with 150,000 reads of 9.7±0.5 Kbp of length sequenced with Minion technology (Oxford Nanopore Technology) per genome. Consequently, genomes were closed with >500x coverage, where detected polymorphisms could unequivocally be assigned to mutations and not be derived from sequencing errors. Genomes sequencings have been submitted to the BioProject database (as Biosamples): PRJNA821238. Annotation of complete genomes was achieved using Prokka v1.14.6. Virulence factors were identified using VFanalyzer, an automatic pipeline analysis to screen FASTA sequences against the VFanalyzer database for known and/or potential virulence factors in a genome.
